# Tight junction between endothelial cells: the interaction between nanoparticles and blood vessels

**DOI:** 10.3762/bjnano.7.60

**Published:** 2016-05-06

**Authors:** Yue Zhang, Wan-Xi Yang

**Affiliations:** 1 The Sperm Laboratory, College of Life Sciences, Zhejiang University, Hangzhou 310058, PR China

**Keywords:** blood vessels, endothelial cells, nanoparticles, oxidative stress, tight junction

## Abstract

Since nanoparticles are now widely applied as food additives, in cosmetics and other industries, especially in medical therapy and diagnosis, we ask here whether nanoparticles can cause several adverse effects to human health. In this review, based on research on nanotoxicity, we mainly discuss the negative influence of nanoparticles on blood vessels in several aspects and the potential mechanism for nanoparticles to penetrate endothelial layers of blood vessels, which are the sites of phosphorylation of tight junction proteins (claudins, occludins, and ZO (*Zonula occludens*)) proteins, oxidative stress and shear stress. We propose a connection between the presence of nanoparticles and the regulation of the tight junction, which might be the key approach for nanoparticles to penetrate endothelial layers and then have an impact on other tissues and organs.

## Introduction

Products related to nanoparticles (NPs) are increasingly growing in number. We can easily find them in, e.g., cosmetics [1], food additives [2], industrial process [3] and, especially, in medical therapy [4] and diagnostics [5]. In light of medical therapy, NPs have shown their extraordinary potential in cancer chemotherapeutics [6] and drug delivery systems [7], which successfully defeat some of the drawbacks in traditional cancer chemotherapy (such as multidrug resistance (MDR) in tumors [6]). They can be modified to enhance the specificity of tumor therapy. Admittedly, no scientist could ignore its prominent latent prospect, but meanwhile numerous researches show their concerns for the adverse effects of NPs, e.g., cytotoxicity [8], unknown effects of its biological distribution [9] and genotoxicity [10].

At the same time, we realize that in most cases the pathway of how NPs enter the human body remains unknown. When we are exposed to NPs, they can enter our body though several pathways (oral administration [11], skin exposure [12], breathing [13], intravenous injection [14]). No matter which way, the NPs will finally reunite in the blood vascular system. Blood vessels function as a transportation pipe for blood, which carries nutrients and other necessary substances, such as hormones. Blood is extensively circulated through those vessels and NPs in the blood may reside on the surface of vessels or go through some barriers, e.g., the blood–brain barrier [15], blood–gas barrier [16] and blood–testis barrier [17], and reach important organs which then may get threatened (brain [18–19], lung [20–21] and testis [22]). In order to reduce possible limitations to the application of NPs, a better understanding of the relationship between the blood vascular system and NPs is very important.

## Review

### What are the side effects of NPs?

The general definition of NPs regards their diameter, that is, NPs are particles between 1 and 100 nanometers in size. Generally, the toxicity of NPs is based on the following mechanisms: oxidative stress, disruption of cell membranes, and unknown effects when they enter organs (Table 1).

**Table 1:** Side effects of NPs.

type of NPs	size	experimental target	treatment	effects	ref.

Au	30 nm	pregnant mice	intravenous injection	emphysema-like changes in lungs	[89]
Au	5.3 ± 1 nm	*Mytilus edulis*	exposed in tanks for 24 h in vivo	cause oxidative stress in *Mytilus edulis* within 24 h of exposure	[42]
Au	22 nm	human oral squamous cell carcinoma (HSC-3)	exposure to extracellular, cytoplasm, and nuclear localized AuNPs and AgNPs	decline in intracellular ATP; reduce HSC-3 cell viability; increased apoptotic population	[90]
Au	10 nm × 39 nm, 10 nm × 41 nm, 10 nm × 45 nm	human lung adenocarcinoma epithelial; human gastric adenocarcinoma cells; mouse embryonic fibroblast; porcine kidney; African green monkey kidney; human normal lung tissue	cell-impedance measurement system; monitoring platform; evaluation of cytotoxic effects with traditional in vitro assays	induce signaling and gene expression to regulate responses in cells	[91]
Ag	5–35 nm	*Paracentrotus lividus*	—	induced dose-dependent developmental defects: delayed development, bodily asymmetry, shortened or irregular arms and behavioral changes	[92]
Ag	—	calf thymus DNA	—	alter the conformation of DNA; bind DNA groove	[93]
Ag	50 nm, 3 μm, 30 μm	osteoclasts (OC) and osteoblasts (OB) cultures	murine osteoclasts (OC) and osteoblasts (OB) exposed to silver particles	dose-dependent cytotoxic effects on OB and OC in vitro	[94]
Cu	15 nm	adult mouse podocytes	treated with different concentrations of nano-Cu	increase oxidative stress; cause podocyte apoptosis	[95]
Cu	204 ± 1 nm	epithelial kidney cells of frog *X. laevis*	exposed to CuO particles of three different sizes	cause DNA damage, decrease cell viability and levels of reduced glutathione (GSH) and eventually cell death	[96]
Cu–Zn alloy	—	human lung epithelial cells	—	induce chromosomal damage and intracellular ROS formation	[97]

For instance, gold NPs can cause serious damage in liver, acute inflammation responses and increase the apoptosis of cells. Interestingly, these results are accompanied by an increasing production of reactive oxidative species (ROS) [23]. Silver NPs also hold the ability to generate ROS, which cause oxidative stress [24]. In addition, they can also induce brain dysfunction and pathology [25] and in some cases have an impact on gene expression in neural cells [26]. CuO NPs reduce cell viability and also cause oxidative stress in human bronchial epithelial cells [27].

### Interaction between NPs and blood circulatory system

The circulatory system or cardiovascular system with its main components blood vessels and the heart, is a crucial organ system providing other organs with, e.g., oxygen and nutrients. Humans and vertebrates have a closed vascular blood systems. The vascular system plays an important role in the transport of materials. NPs enter our body through the dermal system, the ocular system, the respiratory system and the gastrointestinal system. All these organ systems are connected to the vascular system and exert advantageous effects or adverse effects through the vascular system.

It has been proven that lipid NPs and polymer NPs can diffuse into photo-damaged skin through follicles and intercellular spaces [28]. Also, zinc oxide, which is widely used in sunscreen, penetrates into the stratum granulosum of the epidermis [29], which leads us to the assumption that once NPs penetrate through the epidermis, there is a huge possibility to get into the arteriovenous plexus underneath the epidermis. NPs, in the form of multi-walled carbon nanotubes, also cause acute eye irritation after administration [30]. The respiratory system shows its unique role in the uptake of NPs, because inhaled NPs have noxious effects on human health. Apical exposure to NPs (polystyrene nanoparticles, quantum dots, single-wall carbon nanotubes) results in the disruption of the alveolar epithelial barrier, the extent of which also depends on the composition, shape and surface charge of the NPs [31]. Carbon nanotubes also show their capacity to penetrate the pulmonary barrier (air–blood barrier) causing interstitial fibrosis [32]. Zinc oxide NPs take part in inflammatory responses in lung epithelial cells [20]. In the research for oral drug delivery, NPs could be absorbed through the intestine, and bioadhesive polymers could improve this capacity [33]. Reviewing the distributional pathways mentioned above, they all have in common to be connected to the circulatory system. This also holds for respiratory organs, such as the lungs where there is a series of capillaries around the blood–air barrier, several arteries outside the submucosa, and the arteriovenous plexus under the epidermis of the skin.

Since the circulatory system may be the main way of transporting the NPs, we shed light on the distribution of NPs in the circulatory system, and found several studies about NPs side effects on organs such as spleen, liver and kidney. AuNPs distributed mainly in rat livers after a single intravenous administration [34]. Subsequent histopathological changes were also found in liver, spleen and kidney after the intravenous administration with dextran-coated graphene oxide nanoplatelets at a dose of 250 mg/kg and more [35].

### Effects of NPs on blood vessels

In recent researches, NPs interacted differently with blood vessels. Some NPs may be associated with acute vascular physiology effects [36]. Ag NPs may cause some side effects on blood and change the hemostatic function [37]. Si NPs showed also antiangiogenic effects on retinal neovascularization [38]. Several modified NPs were also used in vascular-targeted therapy, which showed their way into endothelial cells and escape from the endosome in vitro [39]. Likewise, while iron oxide NPs are very helpful with their capability for imaging, their toxicity towards vascular endothelial cells cannot be ignored [40]. The same goes also for Au NPs. They can bind to red blood cells to provide great X-ray imaging of the blood flow [41], but there side effects to this such as oxidative stress and toxicity [42].

Additionally, recent studies found a close interaction of NPs with several important barriers (e.g., blood–brain barrier, blood–gas barrier and blood–testis barrier). Plain nanoconjugates and nanosized vehicles are widely utilized as drug delivery tools to cross the blood–brain barrier [43]. Moreover, the translocation of gold nanoparticles through the air–blood barrier was found after a treatment with Asian sand dust, which caused acute inflammation in the lung [44]. Studies also present the effect of NPs on reproductive organs. A study on the toxicity of titanium dioxide nanoparticles showed the ability of NPs to cross the brain–testis barrier and accumulate in mice testes [22]. To further explain the way in which the NPs penetrate the blood–testis barrier, an “elevator hypothesis” has been presented [45].

To investigate the main mechanism of the hazardous effects of NPs toward the cardiovascular system, the anatomical structure of blood vessels need to be fully understood. From the outside to the inside, it can be simply summarized as pericytes and endothelial cells [46], even if distinct types of vessel hold differences in those layer structures. The endothelium cells, a sort of cell shared by all kinds of blood vessels, exhibit multiple interactions with NPs injected into vascular system. Research using endothelial cell cultures in order to quantify the uptake of PLGA NPs showed a concentration-dependent uptake of PLGA [47]. Several NPs (COOH100, PEG100, Methyl100, Lysine100) associate with cells through the ability of protein binding on their surfaces [48]. SiO_2_ causes inflammation and cytotoxicity in human umbilical vascular endothelial cells and these effects are related to the activation of potassium channels [49]. Iron oxide NPs also induce inflammation and malfunction in vascular endothelial systems [50].

In the following, we will present assumptions about how the NPs behave in blood vessels, in particular about (1) NPs pass through the endothelial layer of blood vessels and (2) NPs cause cytotoxicity in surrounding tissues under the endothelial layer.

### Endothelial cells and tight junction

The endothelium provides a thin layer of cells that covers the internal surface of blood vessels and functions as a barrier between the flowing blood in the lumen and the vessel wall. The endothelial cell layer is a shared structure from heart to capillaries, and received attention for its correlation with NPs in blood. Its predominant function is serving as a semi-selective barrier between lumen and peripheral tissue. The malfunction of endothelial cells is often regarded as an early event for the development of atherosclerosis and the damage in endothelial cells may also cause hypertension and thrombosis.

Three kinds of proteins may are particularly for the functioning of the endothelium as a barrier: claudins, occludins and the ZO (*Zonula occludens*) proteins. Because they are the main proteins embedded in the membrane of the tight junction (TJ), the interaction between NPs and the functions of these proteins are of particular interest.

### Claudins

Claudins are a group of proteins with four transmembrane domains and two extremely conservative extracellular loops. The extracellular loop 1 mainly engages in the barrier function, while extracellular loop 2 may narrow the paracellular cleft through the association with itself (less investigated) [51]. The range of its C-terminal end is from 21 to 63 amino acids, which may be associated with the localization of these proteins in the tight junction. However, there are only a limited number of proteins in this family that are expressed in endothelial cells, among those proteins, is claudin-5 which shows its predominant function in the development of chicks [52], and claudin-1, claudin-2, claudin-4, and claudin-5 show higher expression levels than other members during the pregnancy period in the mouse placenta [53].

To find out why NPs have an impact on this incredibly essential protein, more attention needs to be paid to the factors that could affect the structure of claudins and then influence its function in the tight junction. These factors are: (1) phosphorylation and (2) oxidative stress. Tyrosine phosphorylation of claudin-5 caused by the exposure of the endothelium to TGF-β1 is associated with a paracellular permeability of the vascular endothelium [54]. Furthermore, phosphorylation of occludin and claudin-5 caused by RhoK diminishes the barrier tightness in the brain [55]. The serine194 of human claudin-4 was phosphorylated in a human epidermal keratinocyte cell line when a tight junction was formed [56]. Thr203 of claudin-1 was proven to be the essential site to enhance the claudin-1-based TJ function, possibly through its phosphorylation [57]. Recently, both PKA-induced phosphorylation of claudin-5 immunoprecipitates and cAMP-dependent but PKA-independent induction of claudin-5 expression were found in endothelial cells of the porcine blood–brain-barrier, both of which could contribute to the promotion of the TJ function [58]. Claudin-4 requires phosphorylation under certain concentrations of Mg^2+^ to proper localize to the tight junction [59] and it can be phosphorylated by protein kinase C (PKC) at Thr189 and Ser194, which might cause the disruption of the barrier function in ovarian cancer cells [60].

Oxidative stress is also relevant for changes in the tight junction. In an experiment under oxidative stress, the expression levels of claudin-5, occludin, and claudin-2 were decreased, while the expression levels of claudin-4 and claudin-8 increased in the kidney [61]. The oxidative stress induced by H_2_O_2_ in gastric epithelial cells also shows some association with decreased amounts of claudin-4 and claudin-7, and an increased permeability of the tight junction for dextran [62].

### Occludins

Occludins are a family of proteins with four transmembrane domains and five extracellular or intracellular domains. The C-terminus of this family is responsible for the correct assembly of the barrier function and could interact with several proteins for cell survival. The N-terminus corresponds to several properties of the tight junction barrier. The extracellular loops could regulate the permeability of the junction barrier. The expression of occludins has been found in arterial and venous endothelial cells [63]. The regulation of the barrier function based through occludins in endothelial cells can be divided into three parts: (1) phosphorylation, (2) expression level of occludins and (3) shear stress.

Research about the phosphorylation of occludins in rat brain capillaries after embolism showed that an increased level of tyrosine phosphorylation in occludins might play an important role regarding the disruption of the tight junction [64]. Casein kinase Iε is a binding partner of the C-terminus in occludins and could induce the phosphorylation of occludins, which may be important for the regulation of occludins [65]. Other research showed that the tyrosine phosphorylation of occludins diminishes the capacity for binding to ZO-1, ZO-2 and ZO-3, which interact with the C-terminal tail of occludins, and hence shows potential to moderate its function as a barrier [54,66]. Moreover, the phosphorylation caused by RhoK at specific sites of occludins in encephalitic brain tissues might disrupt the function of the TJ barrier in the blood–brain barrier [55]. The expression level of occludins in the endothelial barrier may regulate the barrier function and, in certain cases, may act as the main reason for barrier damage. In a study about the effect of isoflurane exposure on the blood–brain barrier, the researchers found a significant decrease in the expression of occludins after the administration of isoflurane anesthesia. This coincided with a disruption of the hippocampus blood–brain barrier and cognitive dysfunction after exposure [67]. In a study detecting changes of occludins in rat brain capillaries after bile duct ligation researchers suggested a relationship between the time-dependent down-regulation of the occludin expression and a time-dependent increase of superoxide radical levels in the brain [68].

Another factor, shear stress, different from normal stressors, exerts a perpendicular force on the material and shows its influence on blood vessels when blood flows in parallel through the vessel. In the field of angiology, shear stress is embodied in the index of endothelial shear stress, which originates from the friction of flowing blood and is proportionally determined by the viscosity of blood and the spatial gradient of the blood velocity along the wall of the vessels [69]. Low endothelial shear stress stimulates atherogenesis, and the formation and progression of an early artherosclerotic plaque [69]. Results of other researchers showed that a short-term shear stress tends to increase the permeability of endothelial monolayers to LDL. After long-term application, the permeability was reduced to nearly the baseline level, which strongly correlated to the leaky junction around apoptotic cells [70]. Additionally, low shear stress decreased mRNA and protein expression of occludins in endothelial cells and increased mRNA and protein expression of VEDF [71], which may account for the increasing permeability due to the low shear stress.

### ZO proteins

ZO proteins exihibt the ability to link occludins or claudins to the actin cytoskeleton and are generally regarded as TJ scaffold due to several binding sites for TJ proteins and cytoskeletal actin [72]. They also play an indispensable role in the TJ function. Phosphorylation of ZO-1 was detected after 24 h and was further increased after 72 h of patulin treatment, which destructs the TJ function, while the interaction of ZO-1 with claudin-4 decreased after 48 h and was absent after 72 h, showing that the phosphorylation of ZO is also responsible for the disruption of the TJ [73].

#### Hypothetical mechanisms of NPs penetration

**Figure 1:**
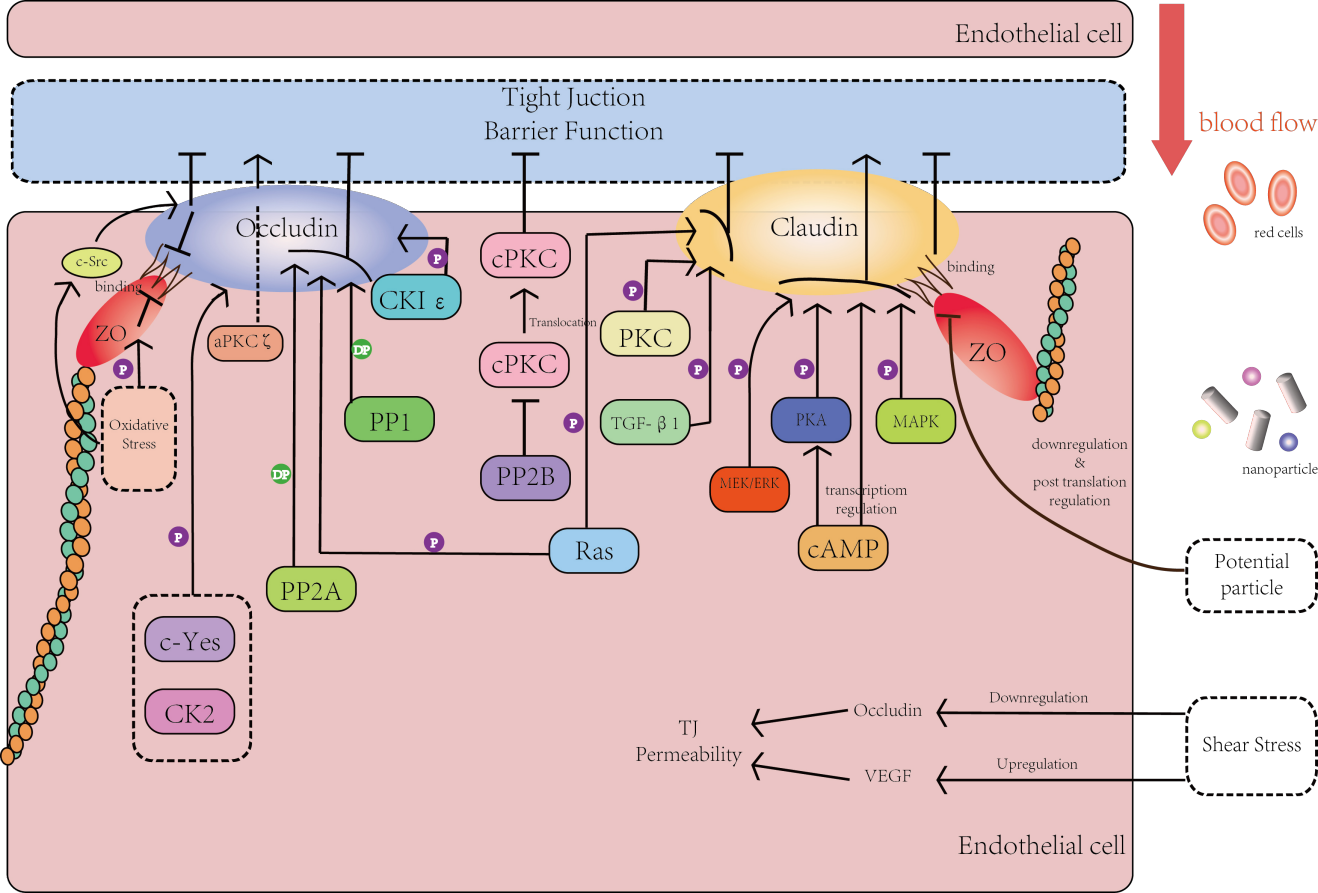
The effects of the phosphorylation process and other processes on the tight junction and its core proteins. For more details see main text.

From what we have discussed above, NPs can penetrate the endothelial monolayer barrier through several pathways. At the beginning of this part, we have to clarify that since the mechanism of the penetration of NPs still remains unclear and the NP materials are very different, here we propose our hypothesis in the form of an analogy. That is to say, we generalize the mechanisms of one specific circumstance in vascular endothelial cells to other kinds of endothelial cells. The hypothetical mechanism includes (1) the phosphorylation reaction of claudins, occludins and ZO proteins, (2) the relationship between oxidative stress and the expression level of claudins and occludins, and (3) shear stress (Figure 1).

Figure 1 mainly describes the interaction between the phosphorylation of key TJ proteins in endothelial cells (occludins, claudins and ZO proteins) and the barrier function of the tight junction. In Figure 1, several important kinases and intermediate products have been expressed by rectangles of different colors. The core proteins of the tight junction (occludins, claudins and ZO proteins) are represented with ellipses of different colors. Lines in Figure 1 stand for intermediate steps: a line with an arrow stands for an activation reaction and a line with a short vertical line stands for a suppression reaction. Moreover, a line with a capital ‘P’ means phosphorylation reaction and a line with the capital letters ’DP’ means dephosphorylation reaction.

On the right side of Figure 1, the arrow in red presents the blood flowing in the vessel. Along with the blood flow, there are blood cells, essential chemicals and NPs in different shapes and sizes. On the left side of Figure 1, there are kinases that have a direct or indirect effect on the phosphorylation or dephosphorylation of core proteins and this way eventually affect the barrier function. Besides the phosphorylation process, the potential influence of shear stress and certain effects on ZO proteins caused by oxidative stress are also shown in Figure 1.

To consider the potential connection between NPs and those crucial proteins, the toxic mechanisms of NPs should be discussed further. So far, we shed light mainly on the effects of whole NPs on cells. Now, the risk of ion release from NPs is to be discussed as well. In an article about the function of several inorganic elements in angiogenesis, Cr, Si, Zn, and Cu ions [74] showed certain connections with several important matters related to the phosphorylation of claudins or occludins. Cr(VI) ions could induce the mutation of several genes in kinase cascades, which are also related to G-protein, Src-kinase and mitogen-activate protein kinases, while mitogen-activated protein kinases are involved in the regulation of the tight junction [75]. In addition, Cr(VI) ions enhanced the tyrosine phosphorylation in human epithelial A549 cells due to H_2_O_2_ and OH radicals caused by Cr(VI) [74]. From this prospective, the unclear mechanism behind the effect of NPs on the tight junction should never be considered to have only one reason, i.e., either the phosphorylation of core proteins or the oxidative stress on those proteins.

As shown above, the three core proteins (claudins, occludins and ZO proteins) play an essential role in the regulation of the TJ barrier function. When considering the way how NPs may have an effect on the regulation, we focus specifically on three mechanisms: (1) direct or indirect interactions with key kinases in the phosphorylation of TJ core proteins; (2) disruption of the oxidative status and oxidative stress around the TJ barrier; (3) changing the shear stress of the blood flow through the physical properties of the NPs.

Recent research showed the interaction between the exposure to NPs and the changing status of signalling pathways based on kinases. In a study using primary osteoblasts as material to evaluate the biocompatibility of 20 nm and 40 nm Au NPs, the Au NPs increased the level of ERK phosphorylation/total ERK [76]. Also, a study of potential mechanisms of Ag-NP toxicity using 8-oxoguanine (8-oxoG) as marker showed the attenuation of both active forms of ERK and AKT protein expression caused by Ag NPs [77]. Another research evaluating the toxicity of quantum dots, found that the production of tumor necrosis factor (TNF)-α and CXC-chemokine ligand (CXCL) 8 in human primary monocytes could be caused by QDs through reactive oxygen species (ROS)- and mitogen-activated protein kinases (MAPKs)-dependent mechanisms [78]. SiO NPs were also found leading to strong ER stress and UPR induction, oxidative stress, activation of MAPK signalling and down-regulation of p53 [79]. Moreover, a study of an important biosynthetic pathway of the B-group vitamins in the genus *Plasmodium*, which is the main malarial parasite, suggested the interaction of Ag NPs with PfThzK (vitamin B1 biosynthetic enzyme 5-(2-hydroxyethyl)-4-methylthioazolekinase from *Plasmodium*), which was non-competitively inhibited (79%) by silver nanoparticles (2–6 nm) [80]. Also, AuNPs have the ability to prevent vascular endothelial growth factor (VEGF)- and interleukin-1 beta (IL-1β)-induced proliferation and migration in bovine retinal pigment epithelial cells (BRPEs) through the suppression of the Src kinase pathway [81]. Silver and gold NPs showed their ability to interact with arginine kinase in *Trypanosoma brucei* by binding to the arginine substrate, which is essential in the transformation between ADP and ATP [82].

In summary, Au NPs, Ag NPs/QDs and SiO_2_ NPs showed the capability to interact with several important kinases and have an impact on biological pathways and activities. In the light of these interactions, if NPs could directly or indirectly affect those kinases referred crucial for the regulation of TJ, they shall in deed have an impact on the barrier function of TJ and as a result cause an increasing permeability or the disruption of TJ.

In addition, NPs cause oxidative stress in different cells and tissues (Table 2). Hence, if NPs find their way into endothelial cells, they may cause oxidative changes in the vicinity of the TJ and thereby influence its function.

**Table 2:** Oxidative stress caused by NPs in different cells and tissues.

type of NPs	size	experimental target	effects	ref.

Fe_2_O_3_	50 nm	human hepatoma Hep G2 cells	concentration-dependent increase of intracellular ROS generation after 12 and 24 h of exposure	[98]
SO-Fe_3_O	44 nm	human lung adenocarcinoma epithelial cell	significant ROS level in cells for the 24 h treatment interval	[99]
ZnO (rod-shape)	15.38 ± 1.47 nm (width); 82.34 ± 14.23 nm (length)	mouse skin epidermal normal cells	time-dependent ROS generation after treatment for 24, 48 and 72 h	[100]
CdS QDs	5–10 nm	mussel hemocytes, mussel gill cells	in hemocytes: increased ROS production with 5 mg Cd/L; in gill cells ROS production was induced with 1.25 mg Cd/L showing time-dependent behavior	[101]
Si/SiO_2_ QDs	3–4 nm	human fetal lung fibroblast cell line	increase of ROS due to the exposure of Si/SiO_2_ QDs	[102]

In terms of shear stress, several NPs have been shown to influence on the shear stress in fluids. A suspension of silica NPs with diameters of 8–25 nm resulted in a surprising shear-thickening under steady shear stress [83], and a suspended silicon dioxide nanoparticle increased the shear viscosity of water around it in equilibrium molecular dynamics simulations [84]. An experimental investigation showed a positive correlation between the concentration of Cu nanoparticles and the viscosity of viscoelastic surfactant solutions [85]. Interestingly, viscoelastic nanofluids containing multiwalled carbon nanotubes also showed non-Newtonian behavior. Their shear viscosity increases with the increase of nanoparticle volume fraction and with a drop in temperature [86]. There is also an interaction between shear stress and cell response to NPs. A study considering cell response to PEGylated poly(dopamine)-coated liposomes under shear stress found that without shear stress, the cellular uptake/association of both PDA-coated liposomes (LPDA) and LPDA-PEG for hepatocytes were quite similar, while myoblasts preferred to internalize/associate with LPDA. However, under shear stress, hepatocytes showed its preference to LPDA after 30 min, while a significant change occurred in myoblasts after 4 h [87]. Recent studies revealed that hydrodynamic conditions influence the endothelial endocytosis of nanocarriers. By using nanocarriers targeted to PECAM-1, the authors found a flow-stimulated endocytosis of nanocarriers through eliciting signaling pathways mediated by RhoA/ROCK and Src family kinases [88]. This further demonstrated the potential connection between phosphorylation and the regulation caused by shear stress.

#### Damage to tissues outside the endothelial layer

To discuss the possible impact of NPs on the cardiovascular system, we shall consider the effects NPs may induce besides the disruption of the endothelial function. For this purpose we will compare the damage NPs cause in other cell lines with the potential risk for cells and tissues beneath the endothelial layer (e.g., pericytes) to be attacked by NPs. Again, oxidative stress plays a major role in this (Table 2). Here, we mainly discuss the oxidative damage NPs may induce in the tissues surrounding the endothelial cells. Interestingly, when injecting nanodrugs intravenously, nanoparticles are mostly released from capillaries, and not from arteries or veins. Therefore, the principal place for the release of nanodrugs is thought to be the capillaries, and while considering the damage towards the surrounding tissue, we pay special attention to pericytes around capillaries [43].

## Conclusion

In this review, we first discussed the wide application of nanoparticles in different fields, e.g., cosmetics, food additives, industry, medical therapy and diagnosis. While nanoparticles are becoming novel and popular materials in numerous scientific areas, their potential side effects are still not clear. Among those potential damages they may cause to the human body, the two most important are cytoxicity and genotoxicity.

While considering nanoparticle effects on human health, we need to know the pathways they use to get into biological systems. Here, we discussed four different entrance pathways: oral administration, skin exposure, breathing intake, intravenous injection. After their passage into the human body, they all face the same barrier, the blood vessel wall, through which they could finally get into tissues and cause different effects in the body. After carefully studying the anatomical structure of blood vessels, the endothelial cells, which function as a barrier on the innermost surface of vessels, become a key point for penetration of NPs and the three core proteins of the tight junction – occludins, claudins and ZO (*Zonula occludens*) proteins – become the main constituents in the interaction between NPs and tight junction.

Additionally, these three core proteins play indispensable roles in the regulation of the tight junction. The phosphorylation of these proteins could promote or impair the barrier function of the tight junction, for instance, they may help to localize those proteins or may cause an increasing permeability of the tight junction. Although recently more research has been carried out showing the potential relationship between the phosphorylation of the TJ proteins and the TJ function, the detailed mechanism for this still remains unclear. Besides, there are also other factors showing the ability to regulate TJ function: oxidative stress and shear stress. A changing oxidative status in endothelial cells shows a certain connection with the expression level of core proteins, which further influence the barrier function of the TJ. Shear stress has a more complex connection with NPs. NPs have a certain possibility to change the viscosity of fluids and in turn the presence of shear stress may change the capacity of cells to uptake nanoparticles.

In conclusion, to discuss the hypothetical mechanism for NPs to penetrate endothelial layers of blood vessels, the phosphorylation of TJ core proteins and its interaction with NPs need to be considered. Moreover, the potential effects NPs may cause on several important kinases might be the key point of this hypothesis. In order to demonstrate the whole influence of NPs on endothelial cells and the tight junction, other factors and their connection with phosphorylation processes have to be considered.

## Future Perspectives

As nanomedicine gradually presents it advantages in medical diagnosis and therapy, the public becomes more and more concerned about their unknown adverse effects on the human body. Many medical researches regard nanoparticles as an extremely suitable material for drug delivery, because of their capability to penetrate several important barriers, e.g., blood–brain barrier, blood–gas barrier and blood–testis barrier. However, this penetration ability may cause other serious effects in the human body, such as damage to other tissues and organs, and unspecific deposition in other parts of the body. Therefore, further investigation have to be done to test hypothetical mechanisms, and to find out efficient methods, which could help to minimize the deleterious impacts caused by NPs, with the utilization of molecular and biochemical tools. This will further increase the safety of nanomedicine applications.
